# Exploring the Future of Cancer Impact in Alberta: Projections and Trends 2020–2040

**DOI:** 10.3390/curroncol30110725

**Published:** 2023-11-18

**Authors:** Darren R. Brenner, Chantelle Carbonell, Dylan E. O’Sullivan, Yibing Ruan, Robert B. Basmadjian, Vickey Bu, Eliya Farah, Shaun K. Loewen, Tara R. Bond, Angela Estey, Anna Pujadas Botey, Paula J. Robson

**Affiliations:** 1 Department of Oncology, University of Calgary, Calgary, AB T2N 4N1, Canada; 2 Cancer Research & Analytics, Cancer Care Alberta, Alberta Health Services, Edmonton, AB T5J 3H1, Canada; 3 Division of Radiation Oncology, University of Calgary, Tom Baker Cancer Centre, Calgary, AB T2N 4N2, Canada; 4 Cancer Strategic Clinical Network, Alberta Health Services, Calgary, AB T2S 3C3, Canada; 5 School of Public Health, University of Alberta, Edmonton, AB T6G 1C9, Canada

**Keywords:** cancer, Canada, projections, trends, cancer incidence, cancer prevalence, cancer mortality, cancer survival, cancer management costs

## Abstract

The impact of cancer in Alberta is expected to grow considerably, largely driven by population growth and aging. The Future of Cancer Impact (FOCI) initiative offers an overview of the present state of cancer care in Alberta and highlights potential opportunities for research and innovation across the continuum. In this paper, we present a series of detailed projections and analyses regarding cancer epidemiological estimates in Alberta, Canada. Data on cancer incidence and mortality in Alberta (1998–2018) and limited-duration cancer prevalence in Alberta (2000–2019) were collected from the Alberta Cancer Registry. We used the Canproj package in the R software to project these epidemiological estimates up to the year 2040. To estimate the direct management costs, we ran a series of microsimulations using the OncoSim All Cancers Model. Our findings indicate that from 2020, the total number of annual new cancer cases and cancer-related deaths are projected to increase by 56% and 49% by 2040, respectively. From 2019, the five-year prevalence of all cancers in Alberta is projected to increase by 86% by 2040. In line with these trends, the overall direct cost of cancer management is estimated to increase by 53% in 2040. These estimates and projections are integral to future strategic planning and investment.

## 1. Introduction

Cancer is a leading cause of morbidity and death worldwide and impacts lives across every world region [1]. In 2020, there were 19.3 million new cancer cases and nearly 10 million cancer-related deaths worldwide [1]. By 2040, the global cancer burden is estimated to increase to 28.4 million projected new cancer cases, representing a 47% increase from 2020 [1]. Higher-developed countries are projected to undergo the greatest absolute increase in incidence by 2040, largely due to population growth and aging [1].

Canada has seen the fastest five-year population growth rate of any G7 nation from 2016 to 2021 at 5.2% [2]. From 2020, the numbers of new cancer cases and cancer-related deaths in Canada are expected to increase by 40% and 44%, respectively [3]. Further, approximately 90% of cancers are expected to be diagnosed in Canadians aged 50 years or more [4]. Compared to Canada’s five-year population growth rate, the province of Alberta follows closely at 4.8% [2].

Cancer has a substantial impact on the lives of Albertans, our community, and our healthcare system. In 2021, Alberta recorded an estimated 22,215 new cancer cases and 6701 cancer-related deaths [5]. Amongst the 31,309 total deaths reported in Alberta in 2021, over 21% were attributable to cancer [6]. Despite the significant improvements in cancer prevention, screening, and care, the overall impact of the cancer burden on Albertans continues to persist and cases are on the rise.

Alberta’s population is growing and aging, which will result in the number of cancer cases rising more than twice as fast as the population. From 1998 to 2021, Alberta’s population increased by 54% which has, in turn, resulted in a 120% increase in new cancer cases [5,7]. With higher proportions of older adults, the risk of being diagnosed with cancer also rises [2]. In Alberta, the leading causes of cancer-related deaths are lung, colorectal, breast, and prostate cancer, which together accounted for 49% of all cancer deaths in 2021 [5]. The occurrence of these cancers is more prevalent at a later age, which becomes increasingly important as Alberta’s population continues to age and increase in size.

There is a need to understand future trends in cancer and their impact in Alberta to develop strategies for appropriate investment and planning to meet Alberta’s future cancer care needs. In response, the Future of Cancer Impact (FOCI) report was initiated by Alberta Health Services’ (AHS) Cancer Strategic Clinical Network (SCN). The report presents an updated summary of key cancer statistics and an overview of how cancer programs and services are organized in Alberta today. Additionally, the report provides commentary on models of cancer care and health equity. It concludes with a series of recommendations for integrated research and investment that will support the appropriate evolution of cancer care in Alberta to better address and prepare for future impacts [8].

In this paper, we present a series of detailed projections and trends of cancer incidence, prevalence, mortality, survival, and management costs in Alberta, Canada. These estimates and projections are integral for future research, planning, and investment purposes.

## 2. Materials and Methods

### 2.1. Study Design and Data Source

A secondary data analysis was formed using data from the Alberta Cancer Registry (ACR). The ACR captures information on all individuals diagnosed with cancer within the province because cancer is a reportable event in Alberta and includes accurate information on the disease stage and time of diagnosis. In addition, the registry houses information on the date and cause of death, along with the last known date of follow-up, which are captured via vital statistics and the population registry. We also retrieved data from the International Cancer Survival Benchmarking (ICBP) SURVMARK-2 online tool [9] to examine historical trends in survival for seven selected cancer sites and compare cancer survival trends in Alberta to the rest of Canada.

All data used in the analyses in this study involved the analysis of anonymized population data that are publicly available via online repositories and did not involve contacting individuals, so consideration and approval by an ethics review board was not required.

### 2.2. Study Measures

#### 2.2.1. Cancer Incidence, Prevalence, and Mortality

We examined cancer incidence (the number of new cases of cancer occurring each year), prevalence (the proportion of people who are alive after being diagnosed with cancer either recently or in the past), and cancer-related mortality (the number of deaths that were a direct cause of cancer). We projected the incidence of all cancers combined, as well as incidence and mortality rates of the 10 most common cancer sites (breast, lung, prostate, colorectal, bladder, melanoma, lymphoma, kidney, thyroid, uterus), separately in Alberta to 2040. We also reported the projection of limited-duration prevalence of all cancers combined, as well as the ten most common cancers in Alberta to 2040. We acquired cancer incidence and mortality data in Alberta from the years 1998 to 2018 from the ACR. One, two-, and five-year limited-duration cancer prevalence data in Alberta from the years 2000 to 2019 by cancer site, sex, and five-year age group were also acquired from the ACR. We calculated absolute increases in their annual incidence, as well as age-standardized incidence rates (ASIRs) and age-standardized mortality rates (ASMRs). The ASIR is defined as the number of cancer cases occurring in a defined population over a specified period of time after adjusting for age. The ASMR is the number of cancer deaths occurring in a defined population over a specified period of time after adjusting for age. We also reported the one-, two-, and five-year prevalence and calculated age-standardized prevalence rates (ASPRs), defined as the proportion of people who are alive after being diagnosed with cancer either recently or in the past over a specified period of time after adjusting for age. We estimated the future incidence, prevalence, and mortality rates using age–period–cohort models implemented using Canproj and the R software [10].

#### 2.2.2. Cancer Survival

We described the age-standardized net survival rates, which is the probability of surviving, at one and five years after diagnosis to examine the historical trends in survival for seven cancer sites (esophagus, colon, rectum, colorectal, stomach, lung, and pancreas) overall, by sex and by age group in Alberta. We also compared the cancer survival in Alberta with the rest of Canada for each cancer site. Using data from the ICBP SURVMARK-2 online tool [9], we obtained age-standardized net survival estimates (with 95% CI) in Alberta for all available cancer sites from 1995–2014 in five-year periods: 1995–1999, 2000–2004, 2005–2009, and 2010–2014. We also calculated the absolute and relative percent change in net survival from 1995–1999 to 2010–2014. The absolute percent change was defined as the absolute difference in net survival between 1995–1999 and 2010–2014. The relative percent change was defined as the difference in net survival between 1995–1999 and 2010–2014, divided by the net survival in 1995–1999, then multiplied by 100%. The one- and five-year net survival in the five-year periods were compared between the seven cancer sites in the overall Albertan population (both sexes, all ages), by sex (male, female), and by age group (15–54, 55–64, 65–74, 75+ years old). The absolute and relative percent change from 1995–1999 to 2010–2014 was compared between cancer sites in the overall population and by sex and age group. Within each cancer site, the one- and five-year net survival in the five-year periods were compared between males and females and between the four age groups. Further, within each cancer site, the absolute and relative percent change from 1995–1999 to 2010–2014 was compared between males and females and between the four age groups. Finally, the one- and five-year net survival in the five-year periods were compared between Alberta and the rest of Canada. These methodological approaches have been previously used in national projections and estimates [3,11].

#### 2.2.3. Cancer Management Costs to the Health System

Lastly, we projected the future costs associated with the management of cancer care, specifically costs to the health system in Alberta for the next 20 years. The estimated cost of cancer management in Alberta presented is based on a study performed in Ontario that reported the estimated main net costs (i.e., the cost difference between patients and matched non-cancer control individuals) by phase of care and sex, which were used to estimate the five-year and lifetime costs [12]. All costs reported are in 2019 Canadian dollars. All estimates refer to the entire Albertan population. We evaluated the cancer management costs for four phases: diagnosis (3 months before diagnosis), initial treatment (from diagnosis date to 6 months after), continuing care (between 6 months after diagnosis and up to 12 months before death), and the terminal phase (up to 12 months before death). The diagnosis phase typically involves testing to establish the cancer diagnosis. Initial treatment includes the primary course of therapy and any neoadjuvant and/or adjuvant therapy. Continuing care encompasses ongoing surveillance and active follow-up treatment for cancer recurrence and/or new primary cancers. The terminal phase captures the intensive services, often palliative in nature, provided at the end of life [12]. Population-based cost estimates were provided for the entire adult population and based on a comprehensive list of costs incurred by the health system, including the cost of diagnostic tests and laboratory services; cost of treatment; cost of all physician services (including primary care, specialists, and other physicians); cost of outpatient prescription drugs covered by the governmental drug benefit programs; and cost of home care, inpatient hospitalizations, and ambulatory care (same-day surgeries and emergency department visits) [12]. We conducted simulation modeling using OncoSim to estimate the cancer management costs [13]. OncoSim is a web-based microsimulation tool developed by Statistics Canada and maintained by the Canadian Partnership Against Cancer (CPAC) that evaluates the impact and value of cancer management strategies [13]. We used the OncoSim All Cancers Model, which simulates the incidence, mortality, and direct cancer management cost of the 27 most prevalent types of cancer in Canada. All model parameters related to incidence, mortality, survival, stages, screening, and population structure have been estimated using reliable data sources (e.g., Canadian Cancer Registry, Statistics Canada) and calibrated and validated against past Canadian cancer data. All these models were based on status quo assumptions, meaning model parameters (such as cancer incidence, stages, survival, screening [breast, cervix, and colorectal], vaccination [cervix], treatment, and population growth) were not modified. For full details on the model parameters, please refer to Appendix 6 in the FOCI report here [8]. To adjust for the anticipated population growth, we also reported the cancer management cost per capita, which is the cost across the projected population. To estimate the cancer management costs for each phase of care, we ran a series of OncoSim simulation models.

### 2.3. Data Analysis

The 2011 Canadian standard population was used for carrying out age standardization for both the historical and projected cancer incidence, mortality, and prevalence rates [14]. Direct age adjustment was applied with the formula $Std\;Rate=\sum_{i}r_{i}\omega_{i}/\sum_{i}\omega_{i}$, in which $r_{i}$ is the age-specific rate of the $i^{th}$ age group and $\omega_{i}$ is the standard population count for that age group.

## 3. Results

### 3.1. Projected Cancer Incidence

**Figure 1:**
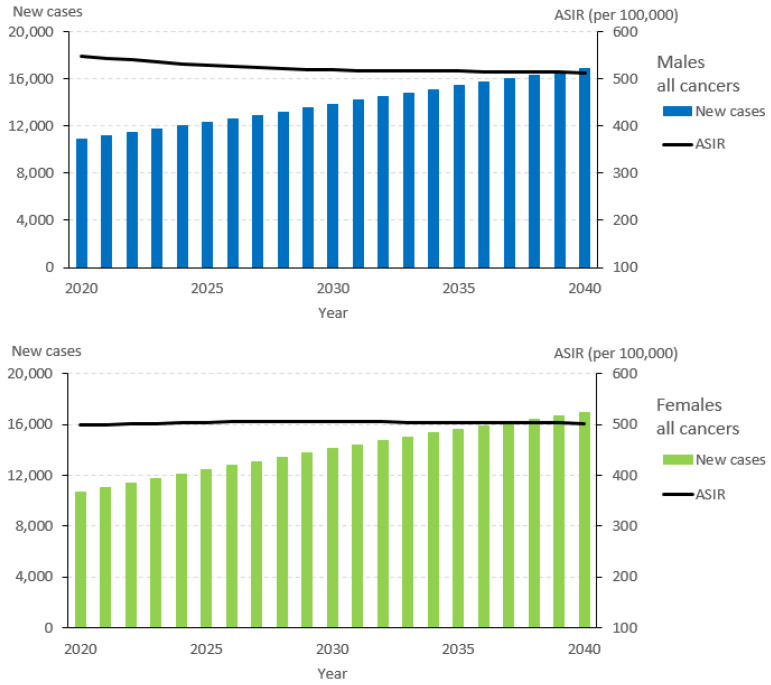
New cases and the age-standardized incidence rate (ASIR) for all cancers, 2020–2040.

The overall annual number of incident cancer cases in Alberta is projected to increase by 29% by 2030 and 56% by 2040, with slightly larger proportional increases for females compared to males (Table 1 and Figure 1). Under this projected scenario, an estimated total of 33,773 incident cancer cases will be diagnosed in 2040, with 16,846 occurring among males and 16,927 among females (Table 1, Figure 1). The 10 most common cancer sites will make up 24,119 (71%) of all cancer cases in 2040 (Table 1). Breast, lung, prostate, and colorectal cancer are the most common cancers in Alberta in 2020 and they are expected to remain so in 2040, comprising 47% of all cancer cases. From 2020 to 2040, the largest absolute increases in annual incidence are anticipated for breast cancer (2255 cases), lung cancer (1019 cases), melanoma (790 cases), prostate cancer (774 cases), and colorectal cancer (699 cases). Among males, the largest absolute increases are projected for prostate cancer (774 cases), lung cancer (655 cases), melanoma (569 cases), and bladder cancer (569 cases). In contrast, the largest absolute increases for females are projected for breast cancer (2255 cases), uterine cancer (461 cases), thyroid cancer (418 cases), and colorectal cancer (407 cases).

While the overall number of incident cases of cancer is projected to increase from 2020 to 2040, the ASIR is projected to decrease by 3% from 524 per 100,000 Albertans to 508 per 100,000 Albertans (Table 2). By 2040, the ASIR is projected to decrease by 6% from 549 per 100,000 Albertans to 513 per 100,000 Albertans for males but increase by 1% from 498 per 100,000 Albertans to 502 per 100,000 Albertans for females (Table 2). The cancer-specific ASIR is projected to decrease for lung (−11%), prostate (−18%), and colorectal (−13%) cancers, but increase for thyroid cancer (25%), kidney cancer (13%), melanoma (11%), and breast cancer (7.8%) (Table 2).

### 3.2. Projected Cancer Prevalence

From 2000 to 2019, the five-year limited-duration prevalence of all cancers in Alberta increased by 105% from 34,482 to 70,687 (Table 3). Breast and prostate cancers account for 19% and 16% of all prevalent cases, respectively, followed by colorectal cancer at 10% and lung cancer at 6%. Among the 10 most commonly diagnosed cancers, thyroid cancer showed the greatest relative increase in the ASPR (from a five-year ASPR of 30 per 100,000 in 2000 to 62 in 2019), whereas lung cancer showed the largest absolute increase in the ASPR (from a five-year ASPR of 83 in 2000 to 120 in 2019). On the other hand, there was a significant reduction in the ASPR for prostate (from a five-year ASPR of 332 in 2000 to 286 in 2019) and colorectal cancers (from a five-year ASPR of 200 in 2000 to 196 in 2019).

**Figure 2:**
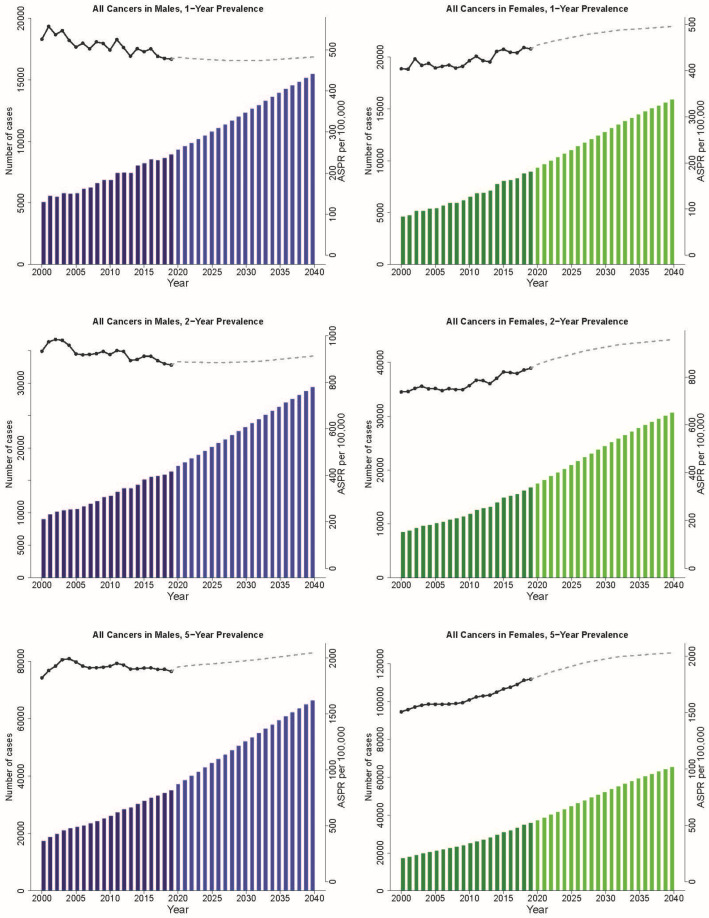
The past and projected cancer 1-, 2-, and 5-year prevalence and the age-standardized prevalence rates of all cancers in Alberta, 2000–2040, by sex.

The projected scenario suggests a gradual increase in the limited-duration prevalence of all cancers combined by 2040 (Figure 2). By 2040, the one-, two-, and five-year prevalence is expected to reach 31,339 cases, 60,004 cases, and 131,660 cases, respectively. Compared to the prevalence rates in 2019, the relative growth in one-, two-, and five-year prevalence rates is projected to be 76%, 82%, and 86%, respectively. In 2040, our projections show that the one-year and two-year ASPR per 100,000 of all cancers in females (496 and 957, respectively) will surpass the ASPR in males (483 and 914), as the gap between the cancer incidence rates in males and females is expected to narrow (as shown in Figure 2). However, the five-year ASPR in females (2033 per 100,000) is expected to be slightly lower than that in males (2049 per 100,000) in 2040.

The current four most prevalent cancers—breast, prostate, colorectal, and lung—will continue to be the most prevalent in 2040). However, colorectal cancer will surpass prostate cancer as the second most prevalent cancer. These four cancers will account for 45% of the five-year prevalence of all cancers in 2040. Similar patterns are also observed for the projected one- and two-year prevalence. Among the four most prevalent cancers in females, the five-year ASPR of breast cancer will continue to rise and be the dominant cancer, whereas the ASPR of lung cancer will decrease to 146 per 100,000 and be surpassed by uterine and colorectal cancers, which are projected to increase, respectively, to 194 and 159 per 100,000 in 2040. Among the four most prevalent cancers in males, the ASPR for prostate cancer is projected to rapidly decrease. In contrast, the ASPRs of colorectal, bladder, and lung cancers will only change slightly.

### 3.3. Projected Cancer Mortality

**Figure 3:**
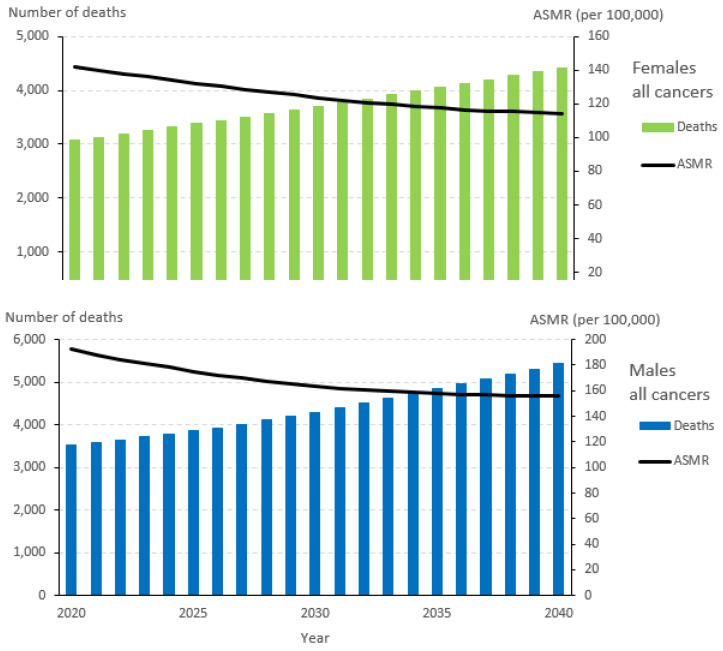
Cancer deaths and age-standardized mortality rates (ASMRs) for all cancers, 2020–2040.

The overall number of annual deaths due to cancer in Alberta is projected to increase by 21% by 2030 and 49% by 2040, with a slightly greater increases for males compared to females (Table 4 and Figure 3). It is estimated by 2040, 9849 Albertans will die from cancer, with 5433 of those deaths occurring among males and 4416 among females (Table 4, Figure 3). Lung, colorectal, breast, and prostate cancers are the leading causes of cancer-related death in Alberta in 2020, and they are expected to remain so in 2040, comprising 45% of all cancer deaths. From 2020 to 2040, the largest absolute increases in annual mortality are projected for lung (358 cases), colorectal (339 cases), prostate (262 cases), breast (233 cases), and bladder (144 cases) cancers. Among males, the largest absolute increases are projected for lung (312 cases), prostate (262 cases), colorectal (219 cases), and bladder (91 cases) cancers. In comparison, the largest absolute increases for females are projected for breast (233 cases), colorectal (120 cases), uterine (106 cases), and bladder (53 cases) cancers.

Although the overall number of cancer deaths is projected to increase by 2040, the ASMR is projected to decline by 20% from 142 in 2020 to 114 in 2040. The ASMR is anticipated to decrease by more than 20% for lymphoma, lung, prostate, and colorectal cancer, remain stable for melanoma (−2%) and thyroid cancer (0%), and increase for uterine cancer (12%). When comparing changes in the ASMR by sex, males are projected to have a larger decrease for bladder cancer and kidney cancer, while females are projected to have a larger decrease for lung cancer, lymphoma, and colorectal cancer.

### 3.4. Historical Trends in Cancer Survival

Among the seven cancers examined, the highest one- and five-year net survival rates from 1995–1999 to 2010–2014 were for colorectal cancer, while the lowest were for pancreatic cancer in both males and females. We observed an increase in the five-year net survival in both males and females for colorectal, esophageal, lung, pancreatic, and stomach cancers. The absolute percent change in five-year net survival from 1995–1999 to 2010–2014 was higher for males than females in colorectal (10% vs. 9%), pancreatic (7% vs. 4%), and stomach cancers (6% vs. 3%). However, for esophageal (12% vs. 4%) and lung (8% vs. 7%) cancers, the absolute percentage change was higher for females than males. Compared to the rest of Canada, Alberta’s one- and five-year net survival rates were similar, except for stomach cancer, which had lower survival rates in Alberta.

### 3.5. Projected Cancer Management Costs to the Health System

**Figure 4:**
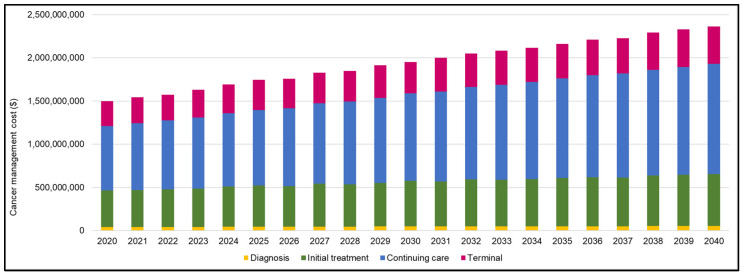
Estimated direct cancer management costs for all cancers combined in 2020 and projected to 2040.

The overall direct cost of cancer management in Alberta is estimated to increase from $1.5 billion (B) in 2020 to $2.3 B in 2040, which represents a 58% increase (Figure 4). All four phases of cancer care are estimated to have relative increases in costs over the projected period. Continuing care is projected to comprise the largest portion of costs, estimated at $0.7 B in 2020 and $1.3 B in 2040, which represents a 71% increase (Figure 4). The second largest estimated cost is for the initial treatment phase, projected to increase from $0.4 B in 2020 to $0.6 B in 2040, a 42% increase. Hematological cancers are estimated to account for the largest estimated direct cost in 2020 and 2024 (Table 5). Together with prostate cancer, hematological cancers account for the largest increase in cost projections for 2040 (69% increase for hematological cancers, $266 M in 2020 to $449 M in 2040, and 72% increase for prostate cancer, $229 M in 2020 to $394 M in 2040) (Table 5). Colorectal and breast cancer are projected to have a 43% and 24% increase in total cancer management costs by 2040, respectively, while lung cancer is projected to increase by 2%. The estimated overall direct cost for cancer management per capita in Alberta was approximately $345 in 2020 and nearly $460 in 2040 (33% increase).

## 4. Discussion

This study presents projections and trends of cancer incidence, prevalence, mortality, survival, and management costs in Alberta from 2020 to 2024. We found the overall number of incident cancer cases and cancer-related deaths are projected to increase considerably by 2040. Similarly, our projections suggest a gradual increase in the limited-duration prevalence of all cancers combined by 2040. We described the historical trends (up to 2014) in the age-standardized net survival rates for seven common cancer sites and observed an increase in the five-year net survival in both males and females for colorectal, esophageal, lung, pancreatic, and stomach cancer. Lastly, it is estimated the overall direct cost of cancer management will increase by 58% from $1.5 B in 2020 to $2.3 B in 2040. The general trends observed in this study may be generalizable to other Canadian provinces and jurisdictions. While Alberta has a younger average population than other provinces, the growth trends are likely to be similar over the next two decades. Furthermore, the historical trends in incidence and survival as well as treatment and outcome patterns are generally similar across provinces.

Projected increases in cancer incidence and cancer-related mortality are driven primarily by an aging population and population growth. Therefore, attention to the unique needs and concerns of older adults with cancer may also be paramount to optimizing outcomes, including frameworks to support screening decisions [15], assessment to support appropriate treatment decision-making, and supportive interventions addressing age-related concerns and the management of comorbidities to promote treatment completion and optimal outcomes [16]. Despite these increases, the ASIRs and ASMRs for some of the commonly diagnosed cancers are declining, which may reflect progress in primary prevention, screening, detection, and treatment. Among the leading cancers, the primary reasons for a decrease in incidence and cancer-related mortality are likely attributable to reductions in the prevalence of smoking (lung and bladder cancers) [17,18], improvements in screening (colorectal cancer), and treatment advances (breast and prostate cancers) [19].

From 2000 to 2019, we observed increases in the ASPRs of thyroid and lung cancers. For thyroid cancer, the increase is mainly due to the increased diagnosis of small papillary cancers of the thyroid, which has emerged as a global issue [20,21]. For lung cancer, because the age-standardized incidence rate has been declining among males and remains steady in females [5], the increase in prevalence is possibly attributable to improved survival. The projection of the five-year ASPR demonstrates that most of the top 10 cancers, except for prostate cancer, are likely to increase. The decreasing five-year ASPR for prostate cancer is mainly attributable to a projection of decreasing cancer incidence. The prostate cancer incidence rate has decreased by about 30% in Canada since 2000 to 2019 [22] and is projected to further decrease by 2040. Additional research into the optimal management of long-term surveillance to maximize outcomes and system efficiency is needed. A projected increasing trend of colorectal cancer prevalence in younger age groups calls for more attention, and there is a need for ongoing monitoring to help us understand more about cancer prevalence in Alberta. Data on sexual orientation and gender identity, race, ethnicity, and socioeconomic status are not currently collected in the ACR and other registries in Canada. Additional investment into linking more comprehensive data sources that capture these factors is essential to evaluate the health disparities and inequalities across the cancer care spectrum. To address this, the Canadian Cancer Society is co-leading a pan-Canadian cancer data strategy with the CPAC to improve data collection, integration, and use. Government funding and investment should prioritize similar initiatives to provide better insight into potential disparities in cancer care, resource access, and outcomes across subpopulations in Canada.

From 1995–1999 to 2010–2014, cancer survival in Alberta was highest for colorectal cancer. The implementation of the fecal occult blood test (FOBT) and fecal immunochemical test (FIT), as part of province-wide colorectal screening in Alberta, has contributed to improvements in survival [23]. Lung cancer was the only cancer type where the net survival was higher for females than males in every five-year period from 1995–1999 to 2010–2014, which may be related to differences in smoking status—including type, volume, and frequency—between males and females. Although daily smoking behavior has declined in Canadian males and females since 1965, males are more likely than females to be daily smokers [24]. Early detection strategies for lung and pancreatic cancers are critical to improving survival, especially since most cases are diagnosed at stage IV with poor prognosis [25]. Greater efforts are needed for cancers where survival remains poor, including lung, esophageal and pancreatic cancer. Continued research efforts are greatly needed to identify potential biomarkers for targeted screening and treatment or for developing prediction tools for risk stratification. Such efforts will better suit patients’ individual needs, particularly when population-based protocols for screening or generic treatments do not work. Analyzing historical cancer trends is valuable for highlighting areas where evidence-based reform is required and laying the foundation for future discussions and planning. There is a clear need to update the data on site-specific cancer survival that are available in the province to identify areas of improvement and priorities. Future funding and investment are vital for additional and sustained innovation in cancer.

The trends in cancer management costs to the health system are affected by several factors including changes in population size, changes in cancer incidence rates, changes in cancer mortality rates, and changes in treatment modalities and the associated costs. The largest portion of cost—and the largest increase in cost—was related to continuing care, which was estimated at $0.7 B in 2020 and $1.3 B in 2040 (71% increase). Continuing care is associated with the expenses incurred for surveillance and active follow-up care, and its increasing cost can be partially attributable to the projected improved survival rate. In addition to increasing costs to the health system, there is a considerable financial burden for patients and families, which is also increasing [26]. Future studies should focus on implementing and evaluating models of care that create value and improve health outcomes. The cost of delays in approvals or gaps in therapeutic coverage should be evaluated as a potential source of system and cost improvement. Investment in studies examining cost-effectiveness of approved therapies, and potential cost savings for biosimilar and other off-patent therapies are also required.

Our study has several strengths. Long-term projections for cancer incidence, prevalence, mortality, and management costs are essential for informing cancer care programs to better target and prioritize prevention initiatives, staffing resources and infrastructure planning, health policy efforts and guiding future research allocations. Identifying cancer sites projected to account for the largest increases in cases and the greatest number of deaths allows primary and secondary prevention programs to be prioritized, treatment resources to be allocated, and research areas to be determined to strategically reduce the impact of cancer incidence and mortality. Projections of cancer prevalence provide useful information for resource planning for diagnostics, treatments, patient care resources, follow-ups and survivorship support [27,28,29] as well as for predicting the demand for future cancer-related health care and social services, and in planning for anticipated challenges [30]. Lastly, understanding costs of cancer care to the Alberta health system and cancer outcomes compared with other provinces is imperative to determine areas of success and areas of opportunity for increased efficiency.

The limitations of methodology in the projections of cancer incidence, mortality, and prevalence should be noted. The cancer incidence, mortality, and prevalence data used to project these statistics to 2040 were taken from the ACR, which is a population-based cancer registry that captures over 99.9% of cancer diagnoses in the province. Nevertheless, the occurrence of certain cancer cases or deaths may be registered in subsequent years, which may influence some of the projected trends. In addition, changes in methods to identify cancer cases over time may impact the number of documented cases, which may influence subsequent projections. We used age–period–cohort models, which rely on assumptions that general historical and recent trends will continue, and these trends may not hold in the real world. Therefore, even though our analyses were based on validated and widely used models, long-term projections involve considerable uncertainty and therefore should be interpreted with caution. Our projections rely on a medium growth rate scenario and pre-date Canada’s new federal immigration targets, which will likely result in greater population growth in Alberta over the coming two decades. It is likely that continued growth trends may lead to an underestimated cancer impact in our analyses [31]. Further, the COVID-19 pandemic, or other future disruptive events, will likely additional unforeseen impacts on patient outcomes that are not accounted for in these models. The findings on cancer survival should also be interpreted with caution. It is likely that the COVID-19 pandemic and public health responses had some impact on the diagnoses and outcomes of cancer in Alberta. Early indications are that for most cancers, these were minimal and not likely to alter the long-term trends and projections included in these results. Net survival is a population-based measure and does not reflect an individual’s survival of cancer. Regarding the projected cancer management costs, all costs are presented as 2019 Canadian dollars. At this time, the OncoSim platform is not able to incorporate modifications of the costs over time (including, for example, inflation). Therefore, the cost for each phase and treatment was fixed through the projection period (2020–2040), which is likely to be an underestimate for many of the associated costs. The parameters of cancer costs in OncoSim were based on the publication of de Oliveira et al. [12], which used data from a cohort of patients from 1997 to 2007. The treatments of many cancer types have evolved rapidly, and continue to evolve. The models are updated with inflationary costs to reflect cost growths. However, rapid increases in costs related to targeted therapies and immunotherapies may be underestimated. Despite the limitations of the methods included here, these projections demonstrate the need to continually monitor trends and costs while applying up-to-date approaches.

## 5. Conclusions

The FOCI report highlights several present and future challenges posed by the increasing incidence, mortality, and cost of cancer in Alberta. Our projections suggest that the impact of cancer will grow substantially with a 53% increase in the numbers of cancer and a 49% increase in the numbers of deaths annually by 2040. These increases will result in considerable jumps in the costs of cancer care. This work serves as a catalyst and a call to action for government, healthcare providers, and policymakers to continue support and investment in research and innovation in Alberta. Investment is needed to develop new approaches to cancer control, coupled with new approaches for evaluation, as it is unlikely that current paradigms will be sustainable under the considerable expected growth rates. The FOCI work also outlines how changes in policy, practice, and the public, combined with continual evaluation, reflection, and investment in new strategies, can reduce the impact of cancer on Albertans, now and in the future.

## Figures and Tables

**Table 1:** Projected cancer incidence overall and for the top 10 sites by sex in the years 2030 and 2040 in Alberta.

	Total					Males					Females				
	2020	2030	Δ%	2040	Δ%	2020	2030	Δ%	2040	Δ%	2020	2030	Δ%	2040	Δ%
All	21,615	27,929	29.2	33,773	56.2	10,919	13,853	26.9	16,846	54.3	10,696	14,076	31.6	16,927	58.3
Breast ^1^	3278	4513	37.7	5533	68.8	NA	-	-	-	-	3278	4513	37.7	5533	68.8
Lung	2608	3259	25.0	3627	39.1	1265	1610	27.3	1920	51.8	1343	1649	22.8	1707	27.1
Prostate	2526	2752	8.9	3300	30.6	2526	2752	8.9	3300	30.6	NA	-	-	-	-
Colorectal	2167	2463	13.7	2866	32.3	1214	1321	8.8	1506	24.1	953	1142	19.8	1360	42.7
Bladder	1083	1471	35.8	1760	62.5	850	1179	38.7	1419	66.9	233	292	25.3	341	46.4
Melanoma	902	1329	47.3	1692	87.6	487	780	60.2	1056	116.8	415	549	32.3	636	53.3
Lymphoma	957	1254	31.0	1458	52.4	537	716	33.3	832	54.9	420	538	28.1	626	49.0
Kidney	783	1158	47.9	1445	84.5	522	802	53.6	1001	91.8	261	356	36.4	444	70.1
Thyroid	679	1013	49.2	1248	83.8	187	279	49.2	338	80.7	492	734	49.2	910	85.0
Uterus	729	968	32.8	1190	63.2	NA	-	-	-	-	729	968	32.8	1190	63.2

^1^ While males are diagnosed with breast cancer, it is rare and not among the top cancer sites for males. As such, these data were omitted. Δ% = Change %.

**Table 2:** Projected age-standardized cancer incidence rates (per 100,000) overall and for the top 10 sites by sex in the years 2030 and 2040 in Alberta.

	Total					Males					Females				
	2020	2030	Δ%	2040	Δ%	2020	2030	Δ%	2040	Δ%	2020	2030	Δ%	2040	Δ%
All	523.68	512.0	−2.2	507.8	−3.0	548.5	519.1	−5.4	513.4	−6.4	498.1	504.7	1.3	502.0	0.8
Breast ^1^	154.1	166.1	7.8	173.0	12.3	-	-	-	-	-	154.1	166.1	7.8	173	12.3
Lung	63.83	56.5	−11.4	50.4	−21.0	65.7	58.9	−10.4	56	−14.8	61.9	54.1	−12.6	44.7	−27.8
Prostate	123.7	102.0	−17.5	101.2	−18.2	123.7	102	−17.5	101.2	−18.2	NA	-	-	-	-
Colorectal	52.88	45.9	−13.2	44.0	−16.7	61.1	50.9	−16.7	48.5	−20.6	44.4	40.8	−8.1	39.5	−11.0
Bladder	28.16	27.1	−3.7	25.1	−10.8	45.1	44.2	−2.0	41.2	−8.6	10.7	9.6	−10.3	8.7	−18.7
Melanoma	21.03	23.4	11.3	23.5	11.6	22.7	27	18.9	29.1	28.2	19.5	19.7	2.1	17.7	−8.3
Lymphoma	23.40	22.9	−2.2	21.2	−9.3	27.2	27	−0.7	24.9	−8.5	19.5	18.7	−4.1	17.5	−10.3
Kidney	18.8	21.3	13.3	22.6	20.2	25.3	29.6	17.0	31.9	26.1	12.1	12.8	5.8	13.1	8.3
Thyroid	15.64	19.5	24.9	21.3	36.5	8.6	10.6	23.3	11.2	30.2	22.9	28.7	25.3	31.7	38.4
Uterus	34.3	36.8	7.3	37.0	7.9	NA	-	-	-	-	34.3	36.8	7.3	37	7.9

^1^ While males are diagnosed with breast cancer, it is rare and not among the top cancer sites for males. As such, these data were omitted. Δ% = Change %.

**Table 3:** Cancer prevalence and age-standardized prevalence rates (number of prevalent cases per 100,000) of the 10 most commonly diagnosed cancers in Alberta in 2000 and 2019.

Sex	Cancer Type	One-Year Prevalence		Two-Year Prevalence		Five-Year Prevalence	
		2000 N (ASPR)	2019 N (ASPR)	2000 N (ASPR)	2019 N (ASPR)	2000 N (ASPR)	2019 N (ASPR)
Males	Prostate	1747 (193.9)	2585 (134.4)	3267 (361.7)	4960 (258.5)	6617 (742.6)	11,323 (598.5)
	Colorectal	650 (69.9)	975 (53.5)	1161 (126.5)	1852 (101.1)	2199 (245.8)	4232 (234.1)
	Bladder	356 (39.1)	691 (40.8)	653 (71.6)	1322 (78.1)	1370 (151.8)	2922 (175.2)
	Lung	372 (40.6)	669 (38.2)	580 (62.6)	1063 (61.0)	860 (92.7)	1816 (103.6)
	Melanoma	203 (19.2)	526 (29.0)	406 (38.5)	953 (52.9)	794 (74.2)	1971 (109.1)
	NHL	190 (16.7)	465 (25.1)	352 (32.1)	836 (44.8)	691 (64.3)	1789 (96.4)
	Kidney	173 (18.0)	374 (18.8)	326 (32.6)	748 (38.1)	633 (62.3)	1691 (86.9)
	Thyroid	60 (4.9)	152 (7.5)	113 (9.1)	309 (15.0)	205 (16.6)	698 (33.9)
	All	5032 (525.4)	8898 (477.1)	8953 (933.9)	16,317 (874.5)	17,310 (1823.2)	34,895 (1879.7)
Females	Breast	1620 (142.1)	3094 (154.8)	3232 (284.5)	5991 (300.0)	6907 (614.0)	13,366 (673.9)
	Lung	344 (31.4)	916 (46.3)	540 (49.8)	1553 (78.2)	844 (78.1)	2693 (136.0)
	Colorectal	537 (50.7)	743 (38.5)	887 (83.1)	1395 (72.4)	1736 (163.5)	3163 (164.3)
	Uterus	286 (26.6)	662 (32.6)	561 (52.1)	1265 (62.4)	1262 (117.4)	2863 (140.8)
	Melanoma	200 (15.7)	429 (21.1)	403 (31.9)	864 (42.7)	895 (70.1)	1802 (89.8)
	Thyroid	154 (11.2)	419 (20.0)	268 (19.8)	855 (40.9)	613 (45.2)	1984 (95.4)
	NHL	165 (14.7)	339 (17.3)	300 (26.7)	637 (32.3)	620 (54.5)	1410 (71.6)
	Kidney	124 (11.0)	182 (9.2)	199 (17.4)	380 (19.1)	415 (36.9)	883 (43.8)
	Bladder	98 (9.1)	179 (9.3)	205 (18.8)	368 (19.1)	475 (43.6)	802 (41.9)
	All	4595 (403.8)	8912 (446.9)	8406 (737.3)	16,703 (836.4)	17,172 (1507.2)	35,792 (1796.7)
Both	Breast	1620 (72.9)	3094 (76.8)	3232 (146.3)	5991 (149.2)	6907 (317.0)	13,366 (336.8)
	Prostate	1747 (87.6)	2585 (64.5)	3267 (163.4)	4960 (124.0)	6617 (331.8)	11,323 (285.5)
	Colorectal	1187 (59.6)	1718 (45.1)	2048 (102.7)	3247 (85.1)	3935 (199.7)	7395 (195.6)
	Lung	716 (34.8)	1585 (42.1)	1120 (54.7)	2616 (69.5)	1704 (83.2)	4509 (119.8)
	Melanoma	403 (16.8)	955 (24.2)	809 (34.0)	1817 (46.3)	1689 (69.9)	3773 (96.1)
	Bladder	454 (22.2)	870 (23.7)	858 (42.0)	1690 (46.1)	1845 (90.5)	3724 (102.7)
	NHL	355 (15.6)	804 (20.8)	652 (29.1)	1473 (38.0)	1311 (58.6)	3199 (82.6)
	Uterus	286 (13.9)	662 (16.2)	561 (27.2)	1265 (31.2)	1262 (61.1)	2863 (70.5)
	Kidney	297 (14.2)	556 (13.6)	525 (24.3)	1128 (27.9)	1048 (48.4)	2574 (63.6)
	Thyroid	214 (7.8)	571 (13.2)	381 (14.2)	1164 (26.8)	818 (30.4)	2682 (61.9)
	All	9627 (449.6)	17,810 (451.9)	17,359 (809.4)	33,020 (836.9)	34,482 (1609.5)	70,687 (1798.4)

Abbreviations: ASPR, age-standardized prevalence rate; NHL, non-Hodgkin lymphoma.

**Table 4:** Projected cancer mortality overall and for the top 10 sites in Alberta by sex in 2030 and 2040.

	Total					Males						Females			
	2020	2030	Δ%	2040	Δ%	2020	2030	Δ%	2040	Δ%	2020	2030	Δ%	2040	Δ%
All	6603	8002	21.2	9849	49.2	3530	4300	21.8	5433	53.9	3073	3702	20.5	4416	43.7
Lung	469	1770	10.9	1954	22.4	832	965	16.0	1144	37.5	764	805	5.4	810	6.0
Colorectal	731	850	16.3	1070	46.4	426	513	20.4	645	51.4	305	337	10.5	425	39.3
Breast ^1^	469	572	22.0	702	49.7	-	-	-	-	-	469	572	22.0	702	49.7
Prostate	393	474	20.6	655	66.7	393	474	20.6	655	66.7	-	-	-	-	-
Bladder	172	228	32.6	316	83.7	120	152	26.7	211	75.8	52	76	46.2	105	101.9
Lymphoma	232	261	12.5	286	23.3	132	154	16.7	177	34.1	100	107	7.0	109	9.0
Kidney	159	197	23.9	250	57.2	58	102	17.6	147	44.1	33	57	35.1	103	80.7
Uterus	114	167	46.5	220	93.0	-	-	-	-	-	13	114	46.5	220	93.0
Melanoma	91	120	31.9	153	68.1	58	76	31.0	96	65.5	33	44	33.3	57	72.7
Thyroid	23	34	47.8	43	87.0	10	15	50.0	18	80.0	57	13	46.2	25	92.3

^1^ While males are diagnosed with breast cancer, it is rare and not among the top cancer sites. As such, these data were omitted. Δ% = Change %.

**Table 5:** Estimated cancer management costs by phase of care for colorectal, lung, breast, prostate, and hematological cancers in 2020 and projected to 2040.

Most Recent Cancer Diagnosed	Phase of Care	2020 ($)	2040 ($)	Increase (%)
Colorectal	Total	122,056,321	175,089,299	43
	Diagnosis	6,201,577	8,727,560	41
	Initial treatment	94,567,346	138,541,923	47
	Continuing care	11,579,055	15,217,625	31
	Terminal	9,708,343	12,602,192	30
Lung	Total	80,062,069	81,711,799	2
	Diagnosis	16,380,639	17,190,802	5
	Initial treatment	31,786,632	30,011,334	−6
	Continuing care	1,898,700	2,001,810	5
	Terminal	29,996,099	32,507,853	8
Breast	Total	102,936,549	127,829,015	24
	Diagnosis	1,229,619	1,602,038	30
	Initial treatment	58,908,315	69,563,079	18
	Continuing care	29,821,787	41,150,979	38
	Terminal	12,976,829	15,512,920	20
Prostate	Total	229,138,720	394,140,016	72
	Diagnosis	2,425,117	3,486,476	44
	Initial treatment	31,956,724	45,942,674	44
	Continuing care	182,577,250	326,191,828	79
	Terminal	11,657,739	18,074,378	55
Hematological	Total	265,712,085	449,081,750	69
	Diagnosis	3,590,863	5,881,751	64
	Initial treatment	43,251,421	70,847,688	64
	Continuing care	162,869,322	275,116,047	69
	Terminal	55,721,320	96,285,800	73

## Data Availability

Data are contained within the article.
